# Exploring association of melanoma-specific Bcl-xL with tumor immune microenvironment

**DOI:** 10.1186/s13046-023-02735-9

**Published:** 2023-07-24

**Authors:** Anna Maria Lucianò, Marta Di Martile, Ana B. Pérez-Oliva, Marica Di Caprio, Maria Laura Foddai, Simonetta Buglioni, Victoriano Mulero, Donatella Del Bufalo

**Affiliations:** 1 Preclinical Models and New Therapeutic Agents Unit, IRCCS Regina Elena National Cancer Institute, Rome, Italy; 2 Departamento de Biología Celular e Histología, Facultad de Biología, Universidad de Murcia, Murcia, 30100 Spain; 3 Instituto Murciano de Investigación Biosanitaria (IMIB) Pascual Parrilla, Murcia, 30120 Spain; 4 Centro de Investigación Biomédica en Red de Enfermedades Raras (CIBERER), Instituto de Salud Carlos III, Madrid, 28029 Spain; 5 Immunohematology and Transfusional Medicine Unit, IRCCS Regina Elena National Cancer Institute, Rome, Italy; 6 Pathology Unit, IRCCS Regina Elena National Cancer Institute, Rome, Italy

**Keywords:** Melanoma, Bcl-xL, Tumor-associated macrophages

## Abstract

**Background:**

Macrophages take center stage in the tumor microenvironment, a niche composed of extracellular matrix and a heterogeneous group of cells, including immune ones. They can evolve during tumor progression and acquire Tumor-Associated Macrophage (TAMs) phenotype. The release of cytokines by tumor and stromal cells, influence the secretion of cytokines by TAMs, which can guarantee tumor progression and influence the response to therapy. Among all factors able to recruit and polarize macrophages, we focused our attention on Bcl-xL, a multifaceted member of the Bcl-2 family, whose expression is deregulated in melanoma. It acts not only as a canonical pro-survival and anti-apoptotic protein, but also as a promoter of tumor progression.

**Methods:**

Human melanoma cells silencing or overexpressing Bcl-xL protein, THP-1 monocytic cells and monocyte-derived macrophages were used in this study. Protein array and specific neutralizing antibodies were used to analyze cytokines and chemokines secreted by melanoma cells. qRT-PCR, ELISA and Western Blot analyses were used to evaluate macrophage polarization markers and protein expression levels. Transwell chambers were used to evaluate migration of THP-1 and monocyte-derived macrophages. Mouse and zebrafish models were used to evaluate the ability of melanoma cells to recruit and polarize macrophages in vivo.

**Results:**

We demonstrated that melanoma cells overexpressing Bcl-xL recruit macrophages at the tumor site and induce a M2 phenotype. In addition, we identified that interleukin-8 and interleukin-1β cytokines are involved in macrophage polarization, and the chemokine CCL5/RANTES in the macrophages recruitment at the tumor site. We also found that all these Bcl-xL-induced factors are regulated in a NF-kB dependent manner in human and zebrafish melanoma models.

**Conclusions:**

Our findings confirmed the pro-tumoral function of Bcl-xL in melanoma through its effects on macrophage phenotype.

**Supplementary Information:**

The online version contains supplementary material available at 10.1186/s13046-023-02735-9.

## Background

A high number of evidence supports the idea that to progress and metastasize, the tumor needs to maintain continuous crosstalk with the surroundings, generating the so-called tumor microenvironment (TME). Even if the progression of cancer has been always described as a multiple steps process in which the only target of genetic and epigenetic changes were the cancer cells, in the last two decades different studies have supported the idea that different components surrounding the tumor are responsible for the tumor behavior [1]. According to that, the establishment and maintenance of TME is an important step for tumor progression, eventually leading to the acquisition of resistance against treatment. Components of TME are tissue resident cells and a large population of cells that are recruited, including immune cells. In fact, even if in the first instance, the immune populations act to reduce cancer cells [2], it is now known that the tumor develops mechanisms through which it escapes from the immune system and, at the same time, modifies the TME in order to support tumor progression. In this process, macrophages play a major role in increasing tumor aggressiveness [3]. Ranging from 30 to 50% of stromal cells, the macrophages with a tumor-promoting role are normally classified as Tumor-Associated Macrophages (TAMs) [3]. Their presence in the TME is associated with poor prognosis in most human cancers [4, 5]. Moreover, TAMs are reported to promote angiogenesis and the maintenance of tumor stem, as well as to interfere with the effectiveness of immunotherapy [6]. Evidence indicate that macrophages exist in a spectrum of states between two extremes, but they are conventionally classified into two phenotypes: the M1 phenotype, also known as “classically activated macrophages” with inherent phagocytic and cytotoxic properties, and the M2, “alternatively activated macrophages” with anti-inflammatory characteristics [7]. Even if it is rare to encounter completely M1- or M2- polarized macrophages, TAMs display a M2 phenotype [8, 9]. The phenotypic switch operated by macrophages is the result of a series of stimuli coming from the TME, and from the tumor cells themselves capable of releasing cytokines, guaranteeing TAMs to sustain a continuous state of inflammation responsible for the pro-tumoral phenotype [8]. The M1 macrophages are normally activated by interferon-γ (IFN-γ) or cytokines like Tumor necrosis factor-α (TNFα) or interleukin (IL)-12, while the M2 phenotype is induced predominantly by IL-4, IL-6, IL-10 and C-C motif chemokine ligand 1 (CCL1) [10]. All these cytokines are released from tumor and stromal cells present in the TME, with consequent induction of different macrophage phenotypes.

TAMs can also affect the nature of the microenvironment of melanoma, among the skin cancers, the one with the highest mortality rate, because of its high ability to metastasize [11]: the infiltration of TAMs correlates with melanoma thickness, increased angiogenesis, and micro-vessel density, through modulation of tumor pro-inflammatory factors [12, 13]. Moreover, the elevated number of TAMs in the melanoma microenvironment is often correlated with a poor prognosis [14]. Thus, macrophages are considered promising therapeutic targets, and their depletion can represent an effective therapeutic intervention in the management of metastatic melanoma [15].

In the last years, our purpose has been to identify potential factors able to induce macrophage polarization and recruitment, focusing our attention on the Bcl-2 family, which has been thoroughly studied due to its multiple canonical and non-canonical functions. In particular, we found that, in addition to the well-known anti-apoptotic role, Bcl-2 expressed in melanoma cells is able to increase tumor aggressiveness [16] and to recruit and polarize macrophages in the M2 phenotype [17]. In this manuscript, we focused our attention on Bcl-xL, which is encoded by the *BCL2L1* gene. Among the different members of Bcl-2 family, Bcl-xL belongs to those proteins that, not only play a pro-survival and an anti-apoptotic role but are, at the same time, capable of inducing tumor aggressiveness, such as tumor invasion/migration, epithelial mesenchymal transition, metastasization and stemness [18]. Several studies in the last years also pointed out the role of Bcl-xL in the regulation of melanoma: overexpression of Bcl-xL in melanoma cells induces secretion of a pattern of factors through which to establish crosstalk with the TME [19–21]. Using *in vitro/in vivo* preclinical models and genetic/pharmacologic approaches, our previous studies supported the concept that Bcl-xL plays a pivotal role in the modulation of properties strictly related to melanoma progression and in the maintenance of cancer stem cell phenotype [19]. Moreover, Bcl-xL regulates in vitro endothelial cell function and in vivo vessel formation in melanoma, through the nuclear factor κB (NF-κB)/IL-8 axis, underling the importance of Bcl-xL as a key regulator of the crosstalk between tumor and neo-vascular endothelial cells [20]. By using in vivo zebrafish model, we previously evidenced the role of Bcl-xL in sustaining and inducing melanoma aggressiveness, via an autocrine pathway involving IL-8 and its receptor C-X-C motif chemokine receptor 2 (CXCR2). Correlation studies of gene expression and survival analysis of melanoma patients also demonstrated that Bcl-xL expression significantly correlates with the expression of IL-8 and other markers of melanoma progression, and that high levels of both Bcl-xL and IL-8 proteins are associated with poor prognosis [21]. Taking in consideration all these functions, specific and non-specific Bcl-xL inhibitors have been developed and several studies reported that interfering with Bcl-xL activity represents a valid approach to enhance the propensity of cancer cells to die [22, 23].

By using in vitro and in vivo models, in this work we described how Bcl-xL overexpressed by melanoma cells, has a high relevance in the macrophage’s polarization and their recruitment, thanks to the release of cytokines such as IL-8 and IL-1β that are able to induce macrophage polarization, and C-C motif chemokine ligand 5 (CCL5, also known as RANTES) that is mainly involved in macrophage recruitment.

## Methods

### Animal models

Zebrafish (*Danio rerio* H.) were obtained from the Zebrafish International Resource Center and mated, staged, raised and processed as described [24]. The *Tg(mfap4.1:tomato)*^*xt12*^ [25], *Tg(tnfa:eGFP-F)*^*ump5*^ [26], *Tg(mpeg1:eGFP)*^*gl22*^ and *Tg(mpeg1:GAL4FF)*^*gl25*^ [27], and *Tg(UAS:NTR-mCherry)*^*c264*^ [28] were previously described. For in vivo invasion assay and macrophage polarization/recruitment, melanoma cells were labelled with 1,1′-di-octa-decyl-3,3,3′,3′-tetra-methyl-indo-carbo-cya-nine perchlorate (DiI, Molecular Probes, Invitrogen, Waltham, MA) and resuspended in a buffer containing 5% fetal bovine serum (FBS) in PBS. Two hundred cells/embryo were then injected in the yolk sac of transgenic zebrafish larvae 2 dpf and maintained for 5 days at 35 °C. Larvae were then analyzed for melanoma cells dissemination and macrophages polarization and recruitment by fluorescence microscopy. Images acquisition was made using a Leica MZ16F fluorescence stereomicroscope and processed using Image J software. The melanoma cell invasion score was calculated as the total number of melanoma cells present in the tail of larvae [29, 30].

Mouse experiments with immunodeficient athymic CD1 nude mice were performed as previously reported [17].

### Cell cultures and treatments

Human melanoma control (Mneo) and Bcl-xL overexpressing stable (Mxl90, Mxl7) cells derived from M14 cell line, A375 and monocytic THP-1 cells, were cultured in RPMI 1640 medium (Euroclone, Milan, Italy), supplemented with 10% inactivated FBS (Gibco, Thermo Fisher Scientific, MA, USA), 1% L-glutamine and 100 µg/mL penicillin/streptomycin (Euroclone). Mneo, Mxl90 and Mxl7 cells were obtained transfecting M14 line with a Bcl-xL expression vector (pcDNA3-Bcl-xL) and an empty vector (pc-DNA3) and cultured with G418 (800 µg/mL, Euroclone) [31].

For siRNA transfection, cells were seeded and, after 24 h, transfected with 20nM pooled siRNA oligonucleotides against CCL5 (si-CCL5), control (si-control) or Bcl-xL (siBcl-xL) (siGENOME SMART pool, DharmaconRNA Technologies, Lafayette, Colorado, USA) by using INTERFERin (Polyplus, Illkirch, France) reagent. Transient transfection for the expression of mutated IKBα (IKBSR) protein was performed using JetPrime (Polyplus). Expression vectors encoding the human IκBSR was kindly provided by Cippitelli M.

For experiments with neutralizing antibodies, cells were treated with human anti-IL-1β (0.2 µg/mL), IL-8 (0.2 µg/mL) or CCL5 (0.2 µg/mL) (R&D Systems, Minneapolis, Minnesota, USA), antibodies for 24 h.

### Monocytes isolation and differentiation

Macrophages have been obtained from (1) THP-1 monocytes using 100 ng/mL phorbol-12-myristate- 13-acetate (PMA, Sigma-Aldrich, San Louis, USA) for 24 h; (2) healthy donor buffy coats, provided by Regina Elena National Cancer Institute, as previously reported [17]. In particular, the PBMC were isolated using Lympholite-H (Euroclone) and plated in RPMI 1640 medium (Euroclone, Milan, Italy), supplemented with 10% inactivated FBS (Gibco), 1% L-glutamine and 100 µg/mL penicillin/streptomycin (Euroclone). After 24 h, monocytes were selected for their ability to adhere to the plate and were incubated for 10 days in RPMI 1640 supplemented with 10% of inactivated FBS and 50 ng/ml MCSF (Peprotech, London, UK) to obtain mature macrophages (monocyte-derived macrophages, M-DM). The, M-DM were stimulated for 24 h in harvesting condition (serum free) and with culture medium (CM) derived from control or Bcl-xL overexpressing melanoma cells. In all experiments, the CM used for stimulating M-DM was normalized to the number of adherent cells. CM from M-DM was collected after 24 h of stimulation with CM from melanoma cells, followed by further 24 h in harvesting condition.

### Cell migration assay

Cell migration assay was performed using a chamber of Transwell (Costar, New York, USA) containing a 5 μm and 3 μm pore polycarbonate membrane for THP-1 and M-DM, respectively. One hundred thousand cells were plated in the upper chamber for 7 h, with the CM derived melanoma cells in the lower chamber. The migrating cells were fixed and stained using the Differential Quick Stain Kit (Dade Behring, Marburg, Germany) and photographed using light microscopy. The quantification was made by counting the migrated cells in 10 images for each condition.

### Elisa and Western blot analyses

ELISA assays for IL-8, IL-1β (Enzo Life Sciences, New York, USA) and CCL5 (RayBiotech (Peachtree Corners, GA, USA) were performed using the CM from M14 melanoma cells, while ELISA for tumor growth factor-β (TGF-β, Fine Test, Whuan, China) was performed using CM from M-DM. Following manufacturer’s instructions, each sample was evaluated in duplicate and protein levels were normalized to the number of adherent cells. Western blot was performed suspending the cellular pellet with modified Ratio-Immunoprecipitation Assay (Tris-HCl 50 mmol/L pH 8, 150 mmol/L sodium chloride, 5 mmol/L EDTA, 0.1% sodium dodecyl sulfate, 1% NP-40, Sigma) placed on ice for 30 min, and centrifuged for 10 min at 4 °C. The supernatants were collected and quantified using the BCA assay kit (Thermo Scientific, Rockford, Illinois, USA). Nuclear and cytoplasmic separation was performed by using the NE-PER Nuclear and Cytoplasmic extraction kit (Thermo Scientific) following the manufacturer’s instructions. Proteins from cellular lysates were fractionated by sodium dodecyl sulfate–polyacrylamide gel electrophoresis, transferred to a nitrocellulose filter, and subjected to immunoblot assays. Immunodetection was performed using antibodies against α-actin (Sigma-Aldrich), Bcl-xL, IKB, p65 and α-tubulin (Santa Cruz Biotechnology, Dallas, Texas, USA), HSP72/73 (Calbiochem, San Diego, California, USA), CCL5 (R&D Systems, Minneapolis, Minnesota, USA), Histone H3 (Cell signaling, Danvers, MA, USA). Anti-rabbit or anti-mouse IgG-horseradish peroxidase-conjugated antibodies (Amersham Biosciences, Freiburg, Germany) were used as secondary antibody. Densitometric analysis was performed using Image J software and normalized with relative α-actin, HSP72/73.

### Human protein array

The Human Angiogenesis Antibody Array C1000 (RayBiotech. Inc., Peachtree Corners, GA, USA) was used according to the manufacturer’s protocol to assess the secretion of more than 40 angiogenic factors into the CM of the different lines. Membranes spotted in duplicate with antibodies against angiogenic factors were blocked with blocking buffer and then were incubated overnight with CM. Next, membranes were washed with wash buffer, incubated with biotin-conjugated antibodies against proangiogenic factors, washed with wash buffer, and incubated with horseradish peroxidase–conjugated streptavidin. The signals on the membranes were detected by chemiluminescence. The intensity of the protein signal (two spots for each protein) was compared with the relative positive signals by densitometric analysis.

### RNA extraction and qRT -PCR

Qiagen RNeasy Mini kit (Qiagen, Hilden, Germany) was used to extract total RNA. Reverse transcription was performed using RevertAid Reverse Transcriptase (Thermo Scientific) kit and Gene-Amp 9700 PCR system (Applied Biosystems, Foster City, California, USA). qRT-PCR was performed using 7900HT Fast Real Time PCR system (Applied Biosystems), using the SYBR green dye detection method. The mRNA levels were normalized using α-actin. The list of primers for each gene was added to the Supplementary Table 1. The results were evaluated by the 2^−ΔΔCt^ method.

### Immunohistochemical (IHC) analysis

IHC analysis of Bcl-xL expression and macrophage polarization or recruitment at the intratumoral or peritumoral area in melanoma xenografts from mice was performed as previously described [17], by using the following antibodies: anti-F4/80 (Thermo Fisher), -CD206 (Abcam, Cambridge, UK) or -Bcl-xL (Cell Signaling) antibodies.

### Statistical analysis

Statistical significance was determined by two-sided paired or unpaired t test for in vitro experiments and by one-way Anova followed by Tukey’s post-test for experiments with Zebrafish models.

The Mann-Whitney test was applied to compare the number of macrophages in the peritumoral or intratumoral area of melanoma tumors from immunodeficient mice. All the statistical analyses were performed by using GraphPad Prism 7.0.

## Results

### Melanoma-specific Bcl-xL induces recruitment and polarization of macrophages in mouse and zebrafish models

**Fig. 1:**
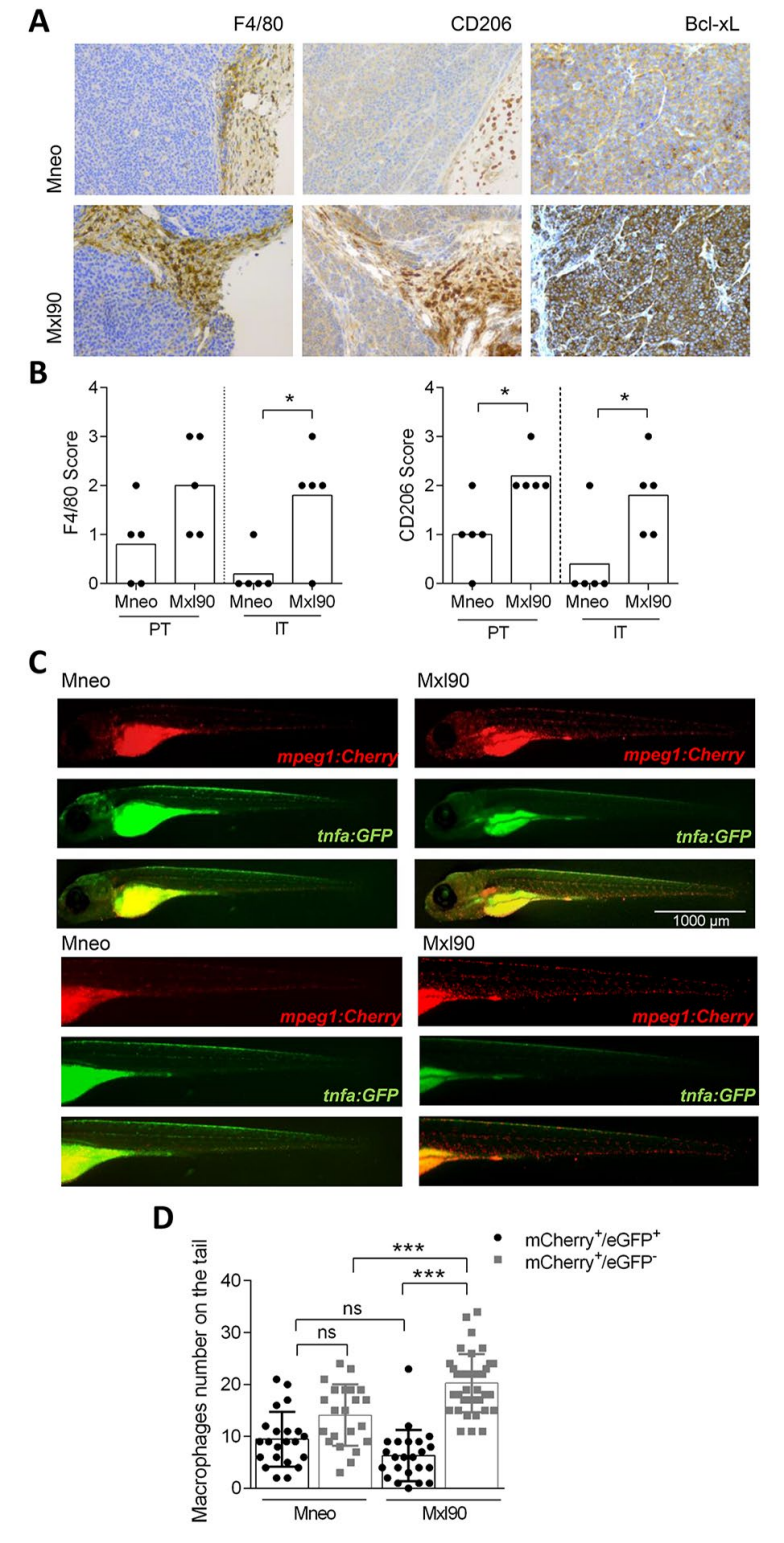
**A**) Representative images of immunohistochemical analysis of F4/80, CD206 and Bcl-xL expression in melanoma control (Mneo) and Bcl-xL overexpressing (Mxl90) tumour-bearing mice, performed 30 days after subcutaneous cell injection (20X magnification), and **B**) relative quantification of peritumoral (PT) and intratumoral (IT) macrophage infiltration by F4/80 and CD206 staining. The results are reported as mean score: score 0, no detectable infiltrate; score 1, low infiltrate; score 2, moderate infiltrate; score 3, high or very high infiltrate. p-values were calculated between control and Bcl-xL overexpressing tumours. **C**) Representative images of Mneo and Mxl90 cells 5 days after injection in the yolk sac of 2 days post fertilization Tg (mpeg1: Cherry/tnfa: GFP) zebrafish larvae, and **D**) relative quantification of M1 (mCherry+/eGFP+) and M2 (mCherry+/eGFP-) macrophages recruitment in the tail.  B,D) Each dot (•) indicates an experimental point, *p < 0.05, ***p < 0.01; ns, no significant differences

Starting from our previous findings demonstrating that melanoma-specific Bcl-2 induces the recruitment and the polarization of macrophages to a M2 phenotype [17], we investigated the possible role exerted by melanoma-specific Bcl-xL in the regulation of macrophage status by using two different animal models, i.e., mouse and zebrafish. Thirty days after subcutaneous injection of control (Mneo) or Bcl-xL overexpressing (Mxl90) M14 melanoma cells in immunodeficient mice, the presence of macrophages infiltration was analyzed by IHC analysis using F4/80 and CD206 antibodies, the former being a specific murine macrophage related marker and the latter a specific M2 macrophage marker. As reported in Fig. 1A,B, a significantly higher number of both intratumoral and peritumoral M2 macrophage recruitment was observed in Bcl-xL overexpressing xenografts when compared to control ones. IHC analysis also confirmed that Bcl-xL protein expression was maintained during in vivo growth (Fig. 1A).

To confirm these data in a different animal model, we took advantage of the use of xenotransplantation assay in zebrafish embryos (Supplementary Fig. 1). Specifically, parental and Bcl-xL overexpressing melanoma cells, were implanted in the yolk sack of zebrafish larvae from the transgenic line *Tg(mpeg1:mCherry*; *tnfa:eGFP*) 48 h post fertilization (hpf), then macrophage polarization on the tail was analyzed 4 days post injection. This transgenic line shows macrophages in red (mCherry^+^) and TNFα-expressing cells in green (eGFP^+^) (*tnfa*) allowing the visualization of M2 macrophages as mCherry^+^/eGFP^−^ and M1 macrophages as mCherry^+^/eGFP^+^ [26]. As observed in mouse models (Fig. 1A,B), a significant increased polarization of macrophages to a M2 phenotype was evidenced in larvae microinjected with melanoma cells overexpressing Bcl-xL when compared to larvae microinjected with parental cells (Fig. 1C,D).

### Melanoma-specific Bcl-xL induces in vitro polarization of human macrophages

**Fig. 2:**
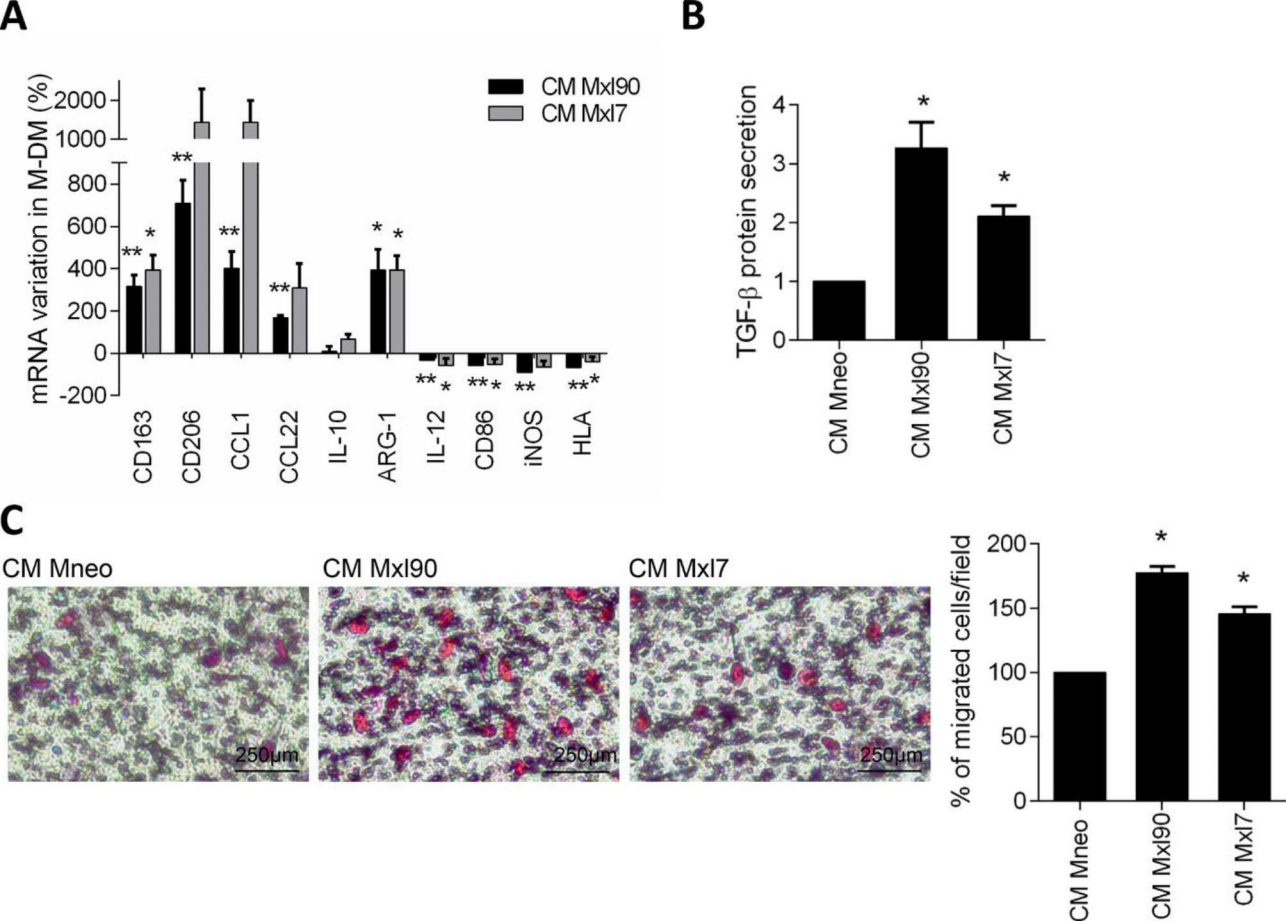
**A**) qRT-PCR analysis of CD163, CD206, CCL1, CCL22, IL-10, ARG-1, IL-12, CD86, iNOS and HLA levels in M-DM after 24 h exposure to conditioned medium (CM) from control (Mneo) or Bcl-xL overexpressing (Mxl90, Mxl7) melanoma cells. The results are reported as percentage of mRNA variation in macrophages exposed to CM derived from Bcl-xL overexpressing cells versus control ones. **B**) TGF-β protein secretion quantified by ELISA essay in M-DM stimulated as reported in A). The results are reported as fold of TGF-β release in M-DM exposed to CM from Bcl-xL overexpressing cells versus control ones. A,B) p-values were calculated between M-DM exposed to CM from Bcl-xL overexpressing cells and those exposed to CM from control cells. **C**) Representative images (left panels) and relative quantification (right panel) of M-DM migration in response to CM from Mneo (CM Mneo), Mxl90 (CM Mxl90) or Mxl7 (CM Mxl7) cells. The quantification was performed by counting the number of migrated cells in at least 10 fields for each condition. The results are reported as percentage of migrated cells/field. p-values were calculated between the percentage of M-DM migrated in response to CM derived from Bcl-xL overexpressing cells, respect to the CM from control melanoma cells. A) The mean ± SEM and B,C) mean ± SD of three independent experiments is reported, *p < 0,05, **p < 0.01

To deeply prove that Bcl-xL was able to induce the polarization of macrophages, M-DM, obtained from healthy donor buffy coats were stimulated with the CM from control (Mneo) or two different Bcl-xL overexpressing (Mxl90, Mxl7) melanoma clones. As reported in Fig. 2A, a significantly higher expression of M2 markers (CD163, CD206, CCL1, CCL22 and ARG-1), paralleled by a significantly lower expression of M1 markers (IL-12, CD86, iNOS and HLA), was observed in M-DM exposed to CM from Bcl-xL overexpressing cells, when compared to CM from Mneo control cells. Accordingly, M-DM stimulated with CM from Bcl-xL transfectants showed increased TGF-β secretion (Fig. 2B). We also evaluated the M2-polarizing ability of CM from A375 (melanoma cell line with high levels of basal/endogenous Bcl-xL) silenced for siRNA control (A375 siCtrl) or for Bcl-xL (A375 siBcl-xL) (Supplementary Fig. 2A). As evident from the Supplementary Fig. 2B, CM from A375 siBcl-xL partially blocked the M2 polarization induced by A375, causing a reduction of CD163, CD206, CCL1 and CCL22 M2 markers.

CM were also used to evaluate their effect on M-DM migration. As reported in Fig. 2C, a significant higher number of migrating cells was detected when M-DM cells were exposed to CM derived from Bcl-xL overexpressing cells and compared to CM from control ones. Similar results were obtained when THP-1 monocytes were exposed to different CM, both in terms of polarization (Supplementary Fig. 2C) and migration (Supplementary Fig. 2D).

### Melanoma-specific Bcl-xL exerts its effect on macrophage polarization and recruitment *via* the release of cytokines

**Fig. 3:**
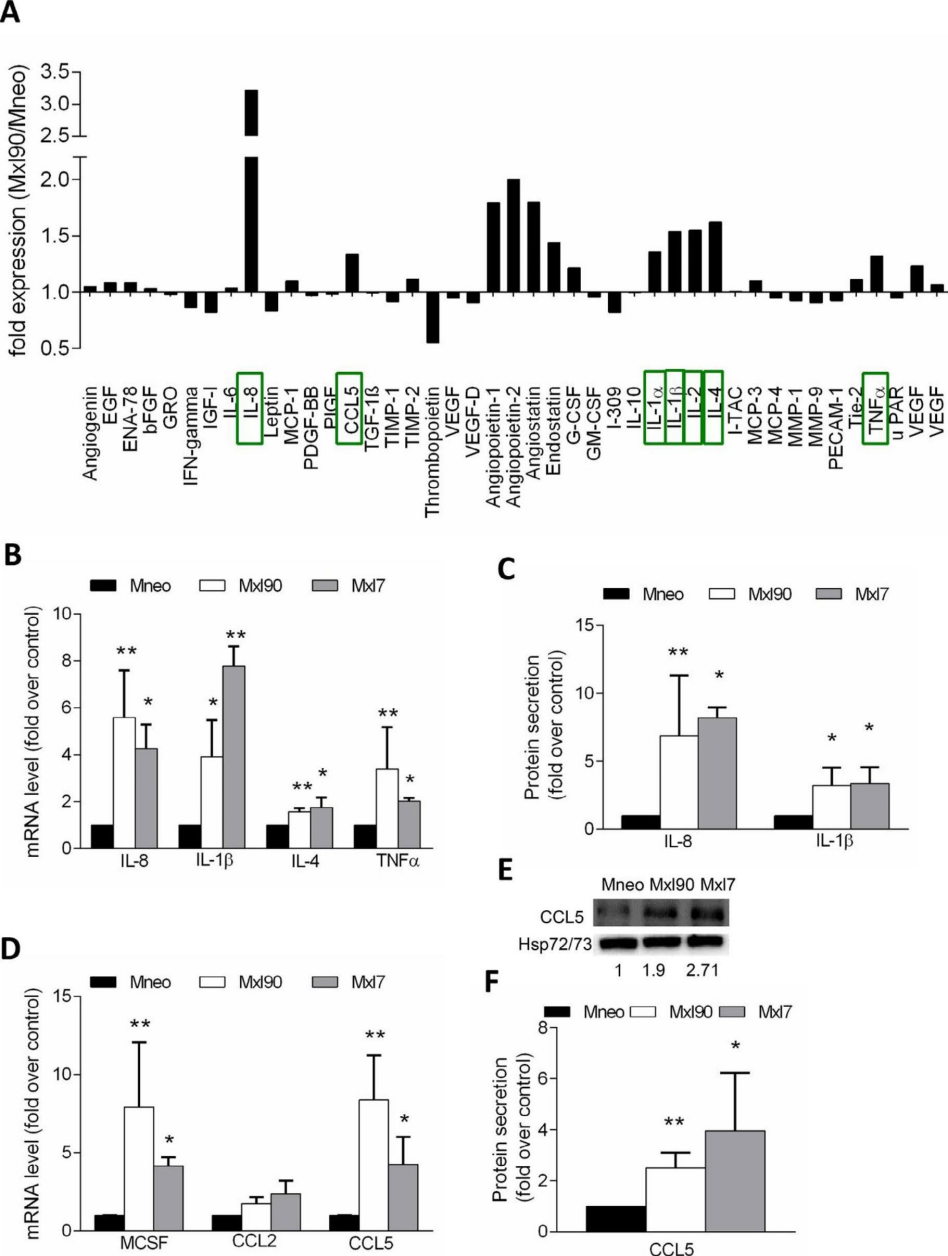
**A**) Quantification of the protein array probed with CM from control (Mneo) or Bcl-xL overexpressing (Mxl90) melanoma cells. Fold expression of Mxl90 relative to Mneo cells is reported. **B,D**) qRT-PCR analysis of **B**) IL-8, IL-1β, IL-4 and TNFα and **D**) MCSF, CCL2 and CCL5 in Mneo, Mxl90 and Mxl7 cells. **C**) IL-8 and IL-1β protein secretion by ELISA in Mneo, Mxl90 and Mxl7 cells. **E,F**) CCL5 protein expression by **E**) Western blot analysis and **F**) ELISA in Mneo, Mxl90 and Mxl7 cells. HSP70 is shown as loading and transferring control. One representative western blot analysis out of two with similar results is reported. The numbers indicate densitometric analysis relative to control. **B-F**) The results represent the average ± SEM of three independent experiments, p-values were calculated between control and Bcl-xL overexpressing cells, *p < 0.05, **p < 0.01

To investigate the possible mechanism underlying the process of macrophage polarization and migration induced by Bcl-xL, we performed a protein array comparing the CM derived from control and Bcl-xL overexpressing M14 cells. As reported in Fig. 3A, the level of several molecules was found up- or down- modulated in the CM from Bcl-xL overexpressing cells, when compared to CM from control ones. Among the up-regulated molecules, we confirmed higher mRNA expression of IL-8, IL-1β, IL-1α, TNFα, IL-2, IL-4 and IL-6, and the recruiting factor CCL5/RANTES by qRT-PCR analysis (Fig. 3B,D and Supplementary Fig. 3A). Higher levels of IL-8, IL-1β and CCL5 proteins were confirmed by western blot and ELISA analyses (Fig. 3C,E,F). Among the other macrophage chemotactic factors beyond CCL5, macrophage-colony stimulating factor (MCSF/CSF1), not present in the protein array, has been also found up-regulated at the mRNA level after Bcl-xL forced expression, while the expression of the chemotactic factor CCL2/MCP-1 was not significantly modulated (Fig. 3D).

**Fig. 4:**
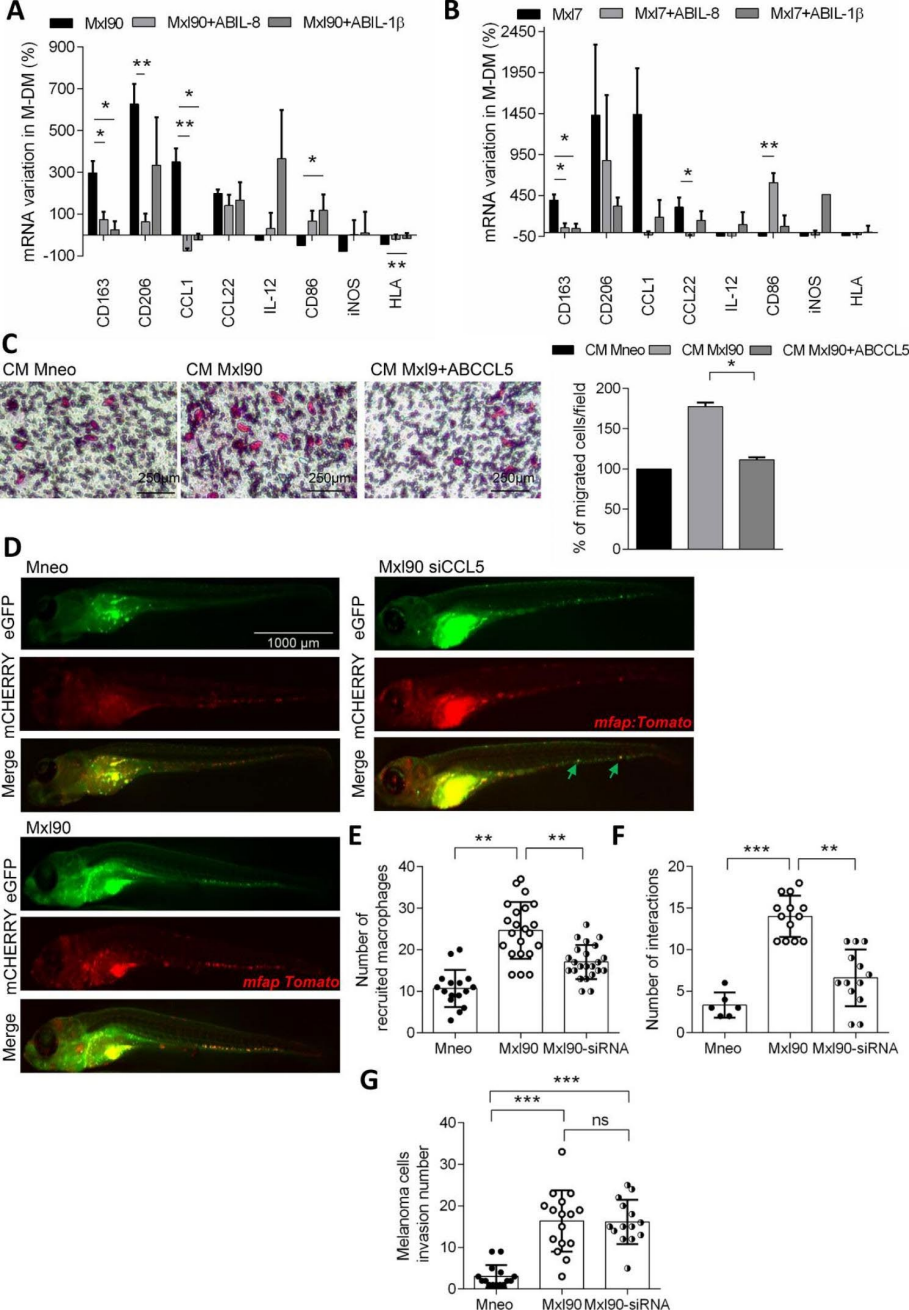
qRT-PCR analysis of CD163, CD206, CCL1, CCL22, IL-12, CD86, iNOS and HLA in M-DM stimulated for 24 h with CM from Bcl-xL overexpressing **A**) Mxl90 and **B**) Mxl7 melanoma cells with or without anti-IL-1β (ABIL-1β) or anti-IL-8 (ABIL-8) blocking antibodies. Percentage of mRNA variation relative to untreated cells is reported. p-values were calculated between Bcl-xL overexpressing cells untreated and treated with antibody. **C**) Representative images (left panels) and relative quantification (right panel) of M-DM migration in response to CM from control (CM Mneo), Mxl90 untreated (CM Mxl90) or treated with the blocking antibody CCL5 (CM Mxl90 + ABCCL5). The quantification was performed by counting the number of migrated cells in at least 10 fields for each condition. p-values were calculated between M-DM migrated cells exposed to CM derived from Bcl-xL overexpressing clones treated and untreated with antibody. **D**) Representative images of Mneo, Mxl90 and Mxl90 cells silenced for CCL5 (Mxl90 siCCL5) stained in green 5 days after injection in the yolk sac of 2 days post fertilization Tg(mfap4:Tomato) zebrafish larvae, and relative quantification of **E**) recruited macrophages, **F**) interaction between melanoma cells and macrophages counted as yellow spots (green arrows), and **G**) invasion score of melanoma cells. p-values were calculated between **E, G**) number of melanoma cells and macrophages recruited in the tail of zebrafish larvae silenced or not for CCL5, **F**) number of interaction between macrophages and melanoma cells silenced or not for CCL5. *p < 0.05, **p < 0.01. ***p < 0.001

Using specific blocking antibodies, we next focused our attention on the role of IL-8 and IL-1β on macrophage polarization, and that of CCL5 on macrophage migration. As reported in Fig. 4A,B, inhibition of IL-8 provoked a significant downregulation of the M2 markers CD163, CD206 and CCL1, while inhibition of IL-1β caused a significant downregulation of the M2 macrophage markers CD163 and CCL1, and a significant increase of the M1 macrophage markers CD86 and HLA.

Importantly, M-DM (Fig. 4C) and THP-1 (Supplementary Fig. 3B,C) cells show a significantly reduced migration when exposed to CM from melanoma cells overexpressing Bcl-xL treated with CCL5 blocking antibody, and compared to cells exposed to CM from Bcl-xL overexpressing cells treated with control siRNA.

We next evaluated the effect of CCL5 silencing by siRNA (Supplementary Fig. 3D) on the ability of Bcl-xL overexpressing melanoma cells to recruit macrophages after microinjection of melanoma cells in the zebrafish line *Tg(mfap4:tomato)*, which has red labelled macrophages. As reported in Fig. 4D-G and Supplementary Fig. 3E, while Bcl-xL overexpressing melanoma cells silenced for CCL5 do not show less ability to invade the tail, inhibition of CCL5 significantly reduced the capacity of macrophages to be recruited at the tumor site, resulting in reduced interactions between invading melanoma cells and macrophages.

### Melanoma-specific Bcl-xL regulates IL-8, IL-1β, MCSF and CCL5 expression through NF-κB

**Fig. 5:**
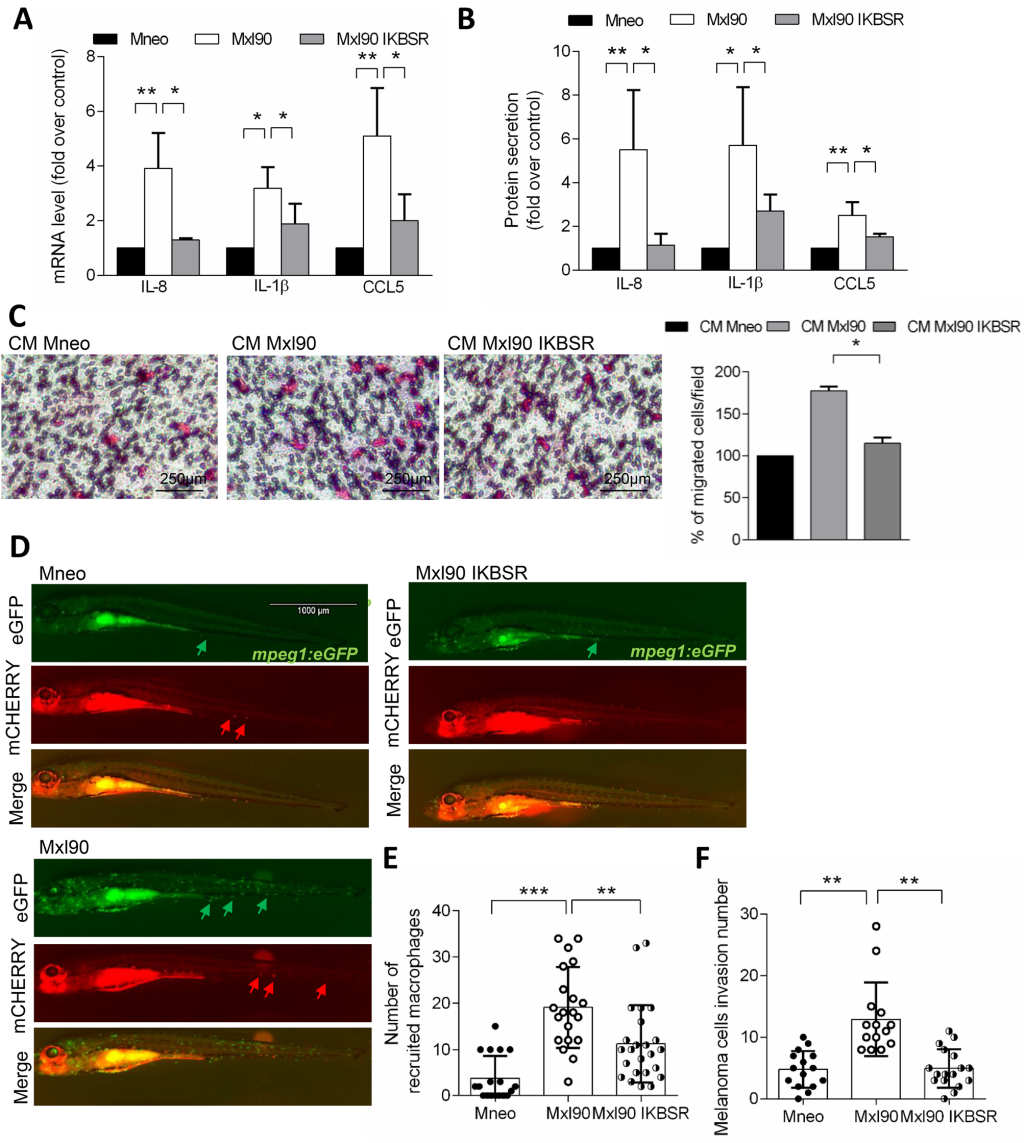
**A**) qRT-PCR analysis and **B**) ELISA of IL-1β, IL-8 and CCL5 expression in control (Mneo) and Bcl-xL overexpressing melanoma cells transfected with IKBSR (Mxl90 IKBSR) or control (Mxl90) vectors. Fold induction relative to Mneo cells and the average ± SEM of three experiments is reported. The average ± SEM of three independent experiments is reported. **C**) Representative images (left panels) and relative quantification (right panel) of M-DM migration in response to CM from Mneo (CM Mneo), Mxl90 (CM Mxl90) and Mxl90 IKBSR (CM Mxl90 IKBSR) cells. The quantification was performed by counting the number of migrated cells in at least 10 fields for each condition. p-values were calculated between the percentage of migrated cells exposed to CM derived from Bcl-xL overexpressing clone transfected with CMV or IKBSR. **D**) Representative images of Mneo, Mxl90 and Mxl90 IKBSR cells (red arrows) 5 days after injection in the yolk sac of 2 days post fertilization Tg (mpeg1: EGFP) zebrafish larvae, and relative quantification of **E**) recruited macrophages (green arrows) and **F)** invasion score of melanoma cells invading the tail. *p < 0.05, **p < 0.01, ***p < 0.001

As up-regulated genes (IL-8, IL-1β, MCSF and CCL5) in Bcl-xL overexpressing melanoma cells have in common that are all regulated by NF-κB [20, 21], we evaluated if this transcription factor was the intermediary between Bcl-xL and the release of these cytokines. To this purpose, we blocked the nuclear translocation of p65 transfecting Bcl-xL overexpressing melanoma cells with IKBSR (Supplementary Fig. 4A,B), the mutated form of IKB, that acts as a NF-κB super repressor [31], and tested the effect on macrophage polarization and migration. We demonstrated that the inhibition of NF-κB was able to reduce IL-8, IL-1β and CCL5 expression at both mRNA (Fig. 5A) and protein (Fig. 5B) levels. We also evidenced a significantly reduced ability of M-DM (Fig. 5C) and THP-1 (Supplementary Fig. 4C) cells to migrate in response to CM from Bcl-xL overexpressing cells transfected with IKBSR compared to cells exposed to CM from control transfected Bcl-xL overexpressing cells.

We next evaluated the effect of NF-κB inhibition on the ability of Bcl-xL overexpressing melanoma cells to invade the caudal region after microinjection in zebrafish larvae of 2 dpf from the transgenic line with labelled macrophages *mpeg1:eGFP*. As reported in Fig. 5D,F, inhibition of NF-κB was able to reduce Mxl90 melanoma cell invasiveness compared to control Mxl90 cells, with a rate of invasion like that of parental Mneo melanoma cells. Strikingly, inhibition of NF-κB in Bcl-xL overexpressing melanoma cells also reduced the number of recruited macrophages, phenocopying the effects of CCL5 inhibition (Fig. 5D,E).

### Depletion of macrophages causes a reduced capacity of melanoma cells to migrate

**Fig. 6:**
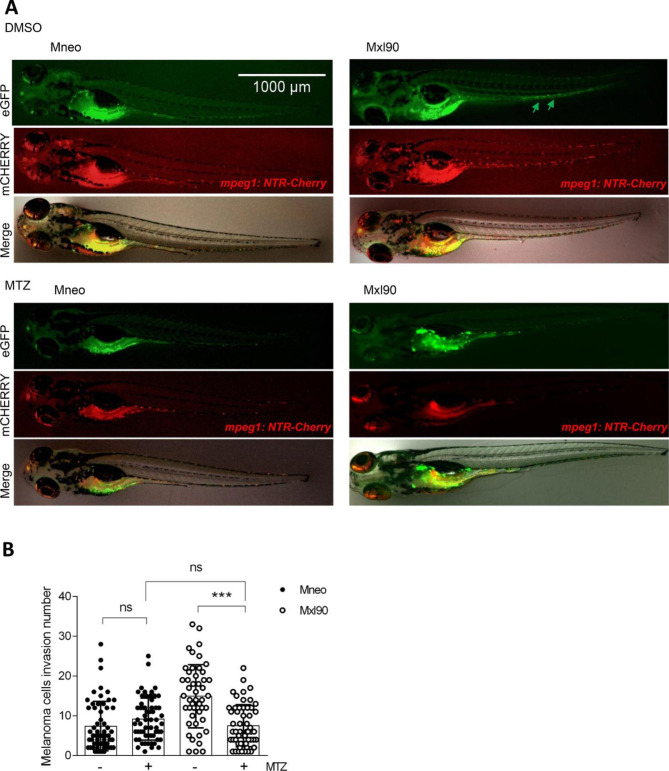
**A**) Representative images and **B**) relative quantification of invasion score of control (Mneo) and Bcl-xL overexpressing (Mxl90) melanoma cells (green arrows) 3 days after injection in the yolk sac of 2 days post fertlization of the mpeg1:GAL4FF, UAS:NTR-mCherry transgenic line expressing bacterial nitroreductase (NTR) fused to mCherry in macrophages, treated with DMSO or 10 mM Metronidazole (MTZ). ***p < 0.001; ns, non-significant

To explore the effect of macrophages ablation on melanoma invasion in the zebrafish xenograft model, we took advantage of the mpeg1:GAL4FF, UAS:NTR-mCherry transgenic line that expresses bacterial nitroreductase (NTR) fused to mCherry in macrophages, allowing the conversion of the prodrug metronidazole (MTZ) in a cytotoxic compound that causes macrophage death [32]. As reported in Fig. 6A,B, macrophage depletion reduced the invasiveness of Bcl-xL overexpressing melanoma cells to the levels observed in Mneo parental cells. As expected, MTZ has no effect on the migration of melanoma cells in *wild type* larvae that did not express NTR (data not shown).

## Discussion

TME has acquired in the last years an important role in the regulation of tumor behavior. Macrophages, which are often closely related to poor prognosis, play a critical role in TME, exerting strong tumor-modulating effects by way of switching their phenotypes [3–7]. Among factors able to recruit and polarize macrophages, we previously identified melanoma-specific Bcl-2 protein [17]. The goal of this study was to evaluate whether the ability to affect macrophage functions was restricted to Bcl-2 or generalized to other anti-apoptotic proteins such as Bcl-xL, and to investigate the molecular mechanism of Bcl-xL-driven macrophages switch. Previous studies from our and other groups evidenced multifaceted roles of Bcl-xL, acting not only as an anti-apoptotic factor, but also being involved in processes strictly related to tumor progression, such as angiogenesis, stemness and epithelial mesenchymal transition [16–21]. In addition, the relevance of Bcl-xL in cancer patients and in particular, in those affected by melanoma, has been well established. In particular, Bcl-xL is expressed at lower levels in nevi, while its expression is increased in advanced melanoma compared with primary ones [16].

By using in vitro melanoma and monocyte cells and two different in vivo models, i.e., mouse and zebrafish, in this study we demonstrated that melanoma-specific Bcl-xL promotes the recruitment of macrophages at the tumor site and induces a pro-tumoral M2 phenotype, though the release of soluble factors. Even if Bcl-2 and Bcl-xL affect macrophages function *via* common factors, from our studies differences emerged between the two anti-apoptotic proteins. In particular, the effect of specific neutralizing antibodies on macrophages functions evidenced that while IL-1β plays a relevant role in both Bcl-2- and Bcl-xL-induced macrophage differentiation, CCL2 and CCL5 were evidenced as chemokines involved, respectively, in Bcl-2 and Bcl-xL ability to induce macrophage recruitment. Our study also demonstrates that Bcl-xL induced expression of IL-8 mediates the effect of Bcl-xL on macrophage polarization. It also indicates that melanoma-specific Bcl-xL could also affect macrophage status through the induction of IL-4 and MCSF. As crosstalk between MCSF and IL-8 has been reported in terms of reciprocal induction in different model systems [33, 34], we can hypothesize also an indirect effect of Bcl-xL on these two factors. Similarly, Bcl-xL induction of MCSF could be indirectly related to TNFα induction previously evidenced by other authors [35].

As we also demonstrated that melanoma specific Bcl-xL induced the expression of Angiopoietins 1 and 2, the ligands of Tie-2 receptor, we cannot exclude that other cytokines, positively regulated by Bcl-xL such as angiopoietin-2, could play a role in macrophage functions, through their ability to enhance tumor-infiltration and proangiogenic or immunosuppressive activities of TIE2-expressing macrophages (TEMs) [36, 37]. Further experiments are needed to validate this hypothesis and to understand the relevance of the angiopoietin 2/Tie-2 pathway in our experimental model.

Several evidences supported the relevance in melanoma progression and response to therapy of chemokines induced by Bcl-xL in this study: we previously reported Bcl-xL-mediated secretion of IL-8 with consequent promotion of aggressiveness [29, 38]; aberrant expression of IL-1β promotes inflammation, invasion, migration and growth as well as the stemness [39–41]; CCL5 is secreted by melanoma cells and is related to tumorigenesis and progression, and affects tumor immune responses [42–44]; and inhibition of MCSF shows anti-tumor efficacy [45, 46].

Our findings are in agreement with those demonstrating (i) IL-1β ability to regulate macrophage functions [47, 48], (ii) the ability of IL-8 neutralization to attenuate the promoting effect induced by GC-MSCs on M2-like macrophage polarization [49], (iii) correlation of CCL5 expressions with infiltration of macrophages in cutaneous melanoma [50]. Once in contact with these cytokines, macrophages undergo a phenotypic and functional switch promoting an anti-inflammatory behavior that regulates malignant progression, through increased angiogenesis and tumor cell invasion [3, 6, 51].

As IL-8 [52], IL-1β [53] and CCL5 [54] are all regulated by NF-κB, we also evaluated whether NF-κB was the common transcription factor through which Bcl-xL induced these chemokines in our models: through genetic approaches inhibiting NF-κB functions we found that all these chemokines are regulated by Bcl-xL in a NF-κB dependent manner. Moreover, interfering with NF-κB activity reduced in vitro and in vivo migration of both macrophages and melanoma cells. In contrast, inhibition of CCL5 production by melanoma cells only affected the recruitment of macrophages, suggesting that these tumor-derived factors can impact the function of resident macrophages in distant sites to sustain tumor aggressiveness. Whatever the outcome, our findings are in agreement with evidence demonstrating the importance of NF-kB in melanoma progression [55, 56], and the relevance of Bcl-2 and Bcl-xL to regulate cancer progression-associated properties through NF-kB. In particular, we previously reported that Bcl-xL and Bcl-2 increase the NF-kB DNA binding activity with a mechanism dependent to IKKα/β and IKBα phosphorylation in glioblastoma and breast cancer [18, 20, 57].

Our data strongly suggest that the previously reported tumor/resistance promoting effect exerted by Bcl-xL [18, 29, 38] could also be related to the induction of different chemokines. We can hypothesize that, in addition to their effect on macrophages phenotype, Bcl-xL-induced cytokines could promote a crosstalk between tumor cells and other stromal cells requiring these cytokines for their function. In particular, Bcl-xL-induced regulation of chemokines could affect the ability of (i) CCL5 to drive NK cell infiltration in melanoma [58] or the response to immunotherapy [43, 59]; *(ii)* MCSF to induce a BRAF inhibitor resistant phenotype [60], or to promote fibroblasts activation or myeloid derived suppressor cells migration [61]; (iii) TNFα to contribute to melanoma invasion/growth and tolerance to MAPK inhibition [62, 63] and to MCSF secretion in macrophages [35], as well as to cooperate with IL-4 in protecting cancer cells from apoptosis [64].

## Conclusions

Our study helps shed light on the different roles played by Bcl-xL in melanoma, and elucidates the comprehensive tumor-microenvironment interaction network, demonstrating a pro-tumoral role of Bcl-xL, and indicating that therapies targeting directly Bcl-xL could be able to weaken the crosstalk between tumor and its microenvironment, blocking its progression.

## Electronic supplementary material

Below is the link to the electronic supplementary material.

Supplementary Material 1

## Data Availability

Data and materials are available upon request to corresponding author.

## Abbreviations

TME: Tumor microenvironment
TAMs: Tumor-Associated Macrophages
IFN-γ: Interferon-γ
TNFα: Tumor Necrosis Factor-α
TGF-β: Tumor Growth Factor-β
IL: Interleukin
CCL1: C-C Motif Chemokine Ligand 1
CXCR2: C-X-C Motif Chemokine Receptor 2
CCL5: C-C Motif Chemokine Ligand 5
NTR: Nitroreductase
MTZ: Metronidazole
M-CSF: Macrophage colony-stimulating factor 1
M-DM: monocytes-derived macrophages
dpf: days post fertilization
hpf: hours post fertilization
