# Supplementary material for: Exploring association of melanoma-specific Bcl-xL with tumor immune microenvironment
Source: J Exp Clin Cancer Res. 2023 Jul 24;42:178. doi: 10.1186/s13046-023-02735-9 (PMC10364435; doi:10.1186/s13046-023-02735-9)
Supplement: Supplementary file 1 — Supplementary Material 1 [file 13046_2023_2735_MOESM1_ESM.pdf]

SUPPLEMENTARY

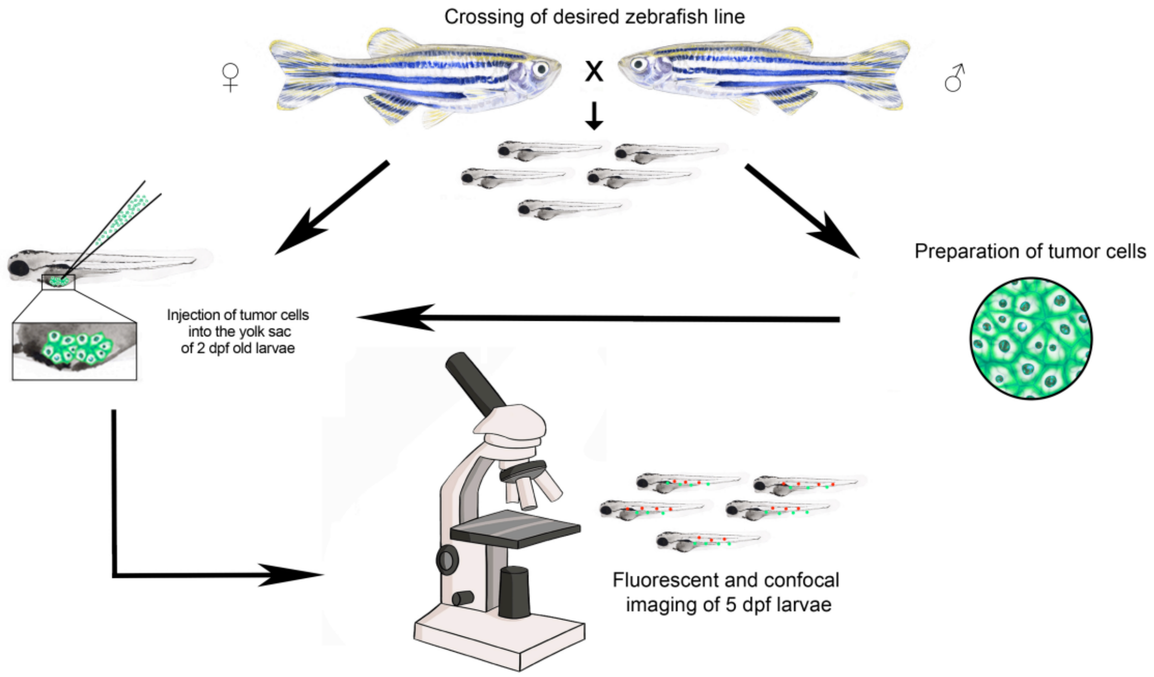

Figure 1. Schematic representation of melanoma cells microinjection in zebrafish larvae. Once obtained the larvae crossing zebrafish lines of interest, melanoma cells were prepared to be injected, marking them with a fluorescent stain. At 2 days post fertilization (dpf), the larvae were injected into the yolk sac, and melanoma cell invasion was analysed at 5 dpf with fluorescent microscopy.

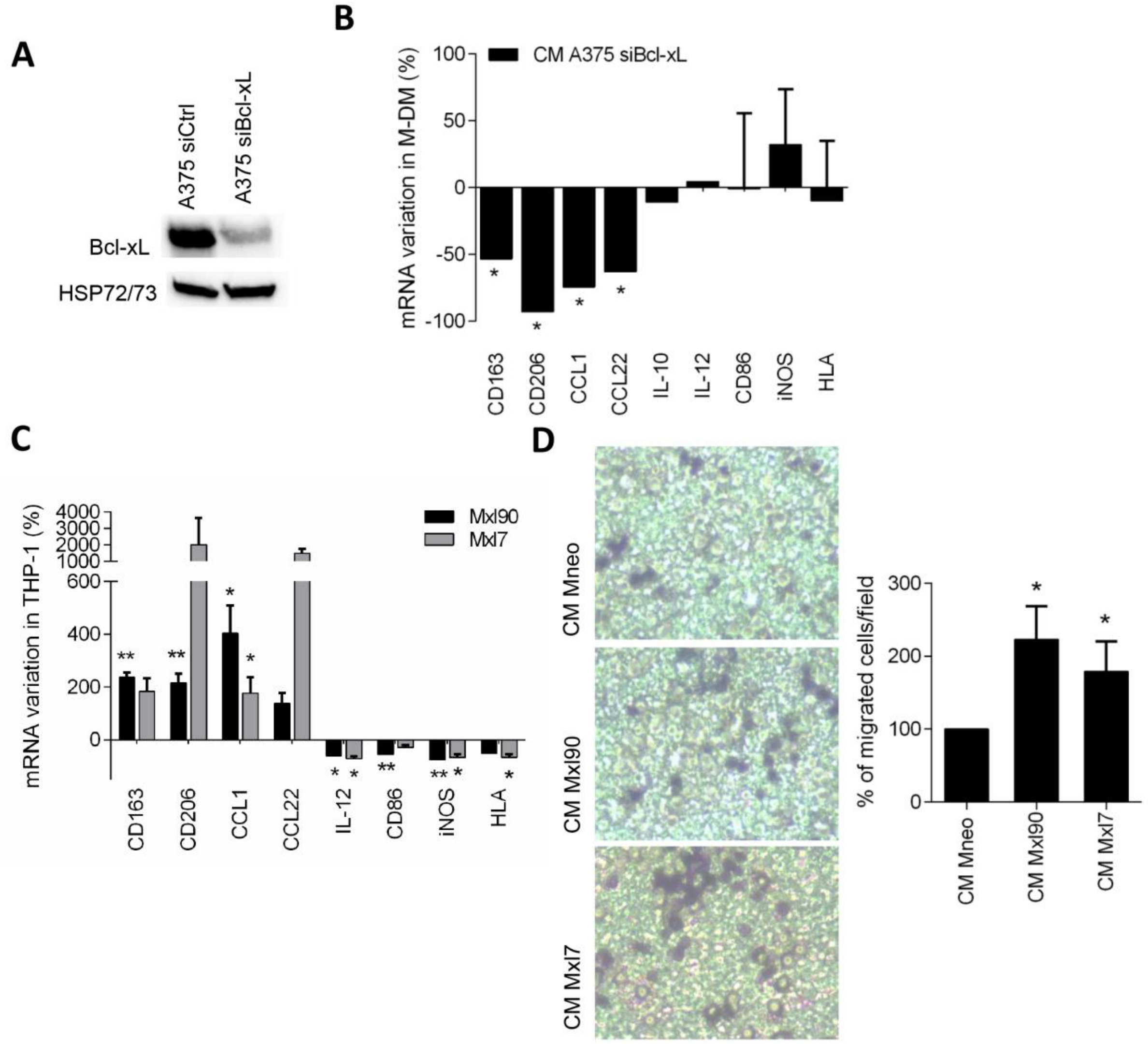

Figure 2. A) Western Blot analysis of Bcl-xL levels in A375 cells silenced with siRNA control (siCtrl) or against Bcl-xL (siBcl-xL). HSP72/73 is shown as loading and transferring control. One representative western blot analysis out of two with similar results is reported. B) qRT-PCR analysis of CD163, CD206, CCL1, CCL22, IL-10, IL-12, CD86, iNOS and HLA levels in M-DM after 24 hours exposure to CM from A375 siCtrl or A375 siBcl-xL cells. C) qRT-PCR analysis of CD163, CD206, CCL1, CCL22, IL-12, CD86, iNOS and HLA levels in THP-1 cells after 24 hours exposure to conditioned medium (CM) from control (Mneo) or Bcl-xL overexpressing (Mxl90, Mxl7) melanoma cells derived from M14 cell line. D) Representative images (left panels) and relative quantification (right panel) of THP-1 cell migration in response to CM from Mneo (CM Mneo), Mxl90 (CM Mxl90) or Mxl7 (CM Mxl7) cells. The quantification was performed by counting the number of migrated cells in at least 10 fields for each condition. The results are reported as percentage of migrated cells/field. B,C) The results are reported as percentage of mRNA variation in macrophages exposed to CM derived from B) A375 siBcl-xL versus A375 siCtrl cells and from C) Bcl-xL overexpressing cells versus control ones. C,D) The mean±SEM and D) mean±SD of three independent experiments is reported. *p<0,05, **p<0.01.

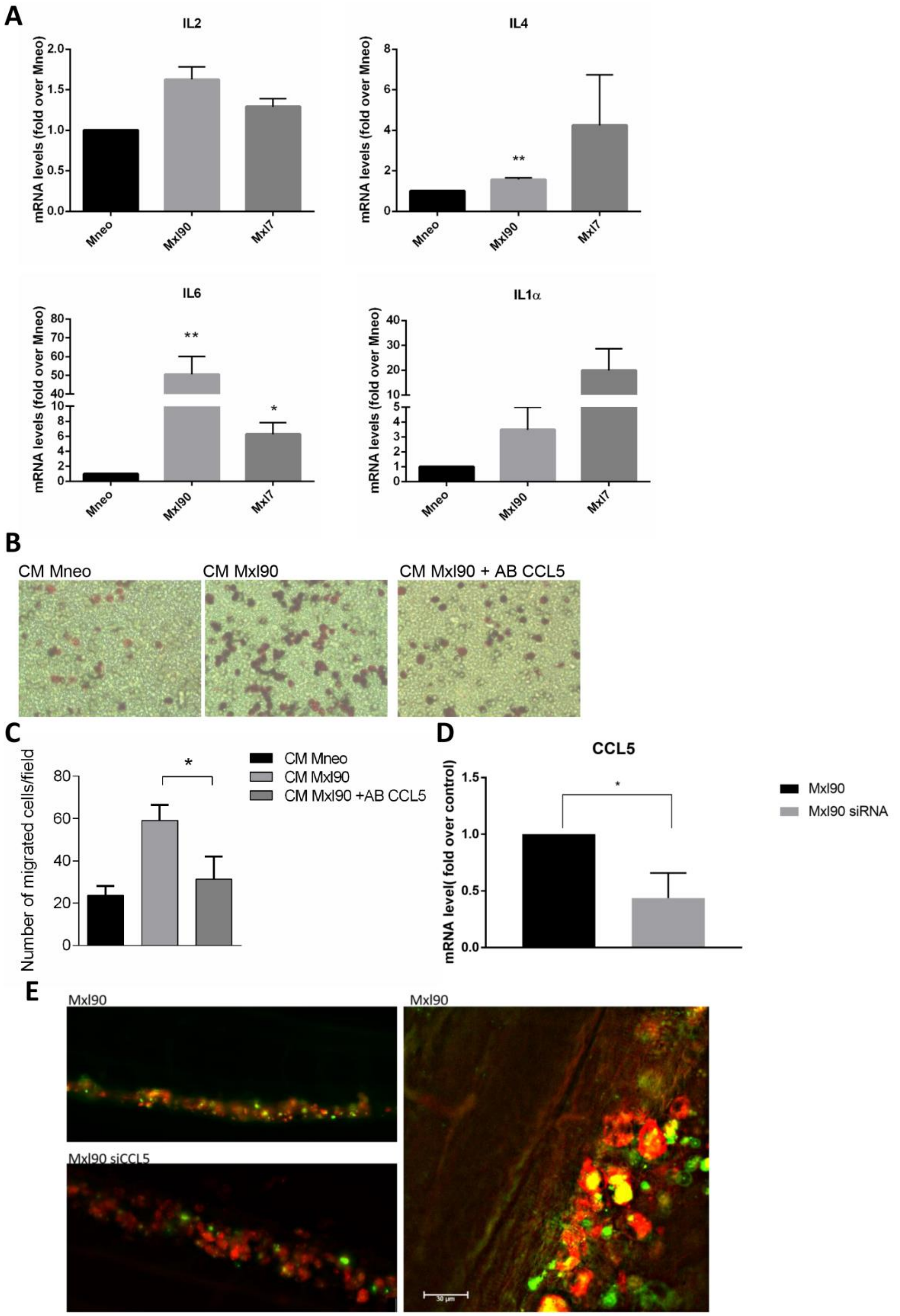

Figure 3. A) qRT-PCR analysis of IL2, IL4, IL6 and IL1α in control (Mneo) or Bcl-xL overexpressing (Mxl90, Mxl7) melanoma cells derived from M14 cell line. The results are reported as % of mRNA variation in Mxl90 and Mxl7 cells compared to the Mneo control cells. B) Representative images and C) relative quantification of THP1 cell migration in response to conditioned medium (CM) from Mneo (CM Mneo), Mxl90 (CM Mxl90) untreated or treated with anti-CCL5 antibody (CM Mxl90+ABCCL5). The quantification was performed by counting the number of migrated cells in at least 10 fields for each condition. D) qPCR of CCL5 expression in Mxl90 cells after the silencing with siRNA. E) Representative scanning laser confocal images of macrophage interactions with control and CCL5-silenced Mxl90 cells.

The left panel represents a major lens magnification of the larvae microinjected with Mxl90 cells. A,D) The mean±SEM and C) mean±SD of three independent experiments is reported. *p<0.05, **p<0.01.

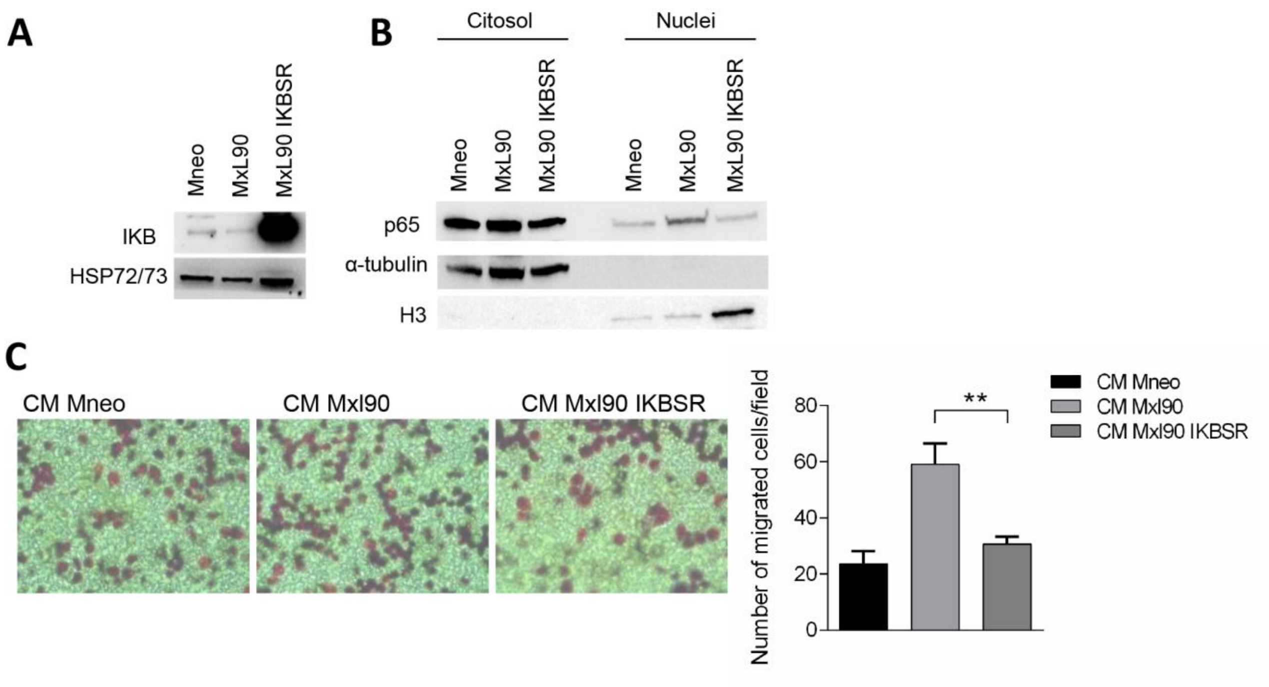

Figure 4. A,B) Western Blot analysis of A) IKBα in the total extract and B) p65 in nuclear and cytosol extracts in control (Mneo) or Bcl-xL overexpressing (Mxl90) melanoma cells derived from M14 cell line, and in Mxl90 IKBSR cells. A) HSP72/73 is shown as loading and transferring control. B) α-tubulin and histone H3 are shown as loading, transferring and cytoplasmic/nuclear purification control. One representative western blot analysis out of three with similar results is reported. C) Representative images and relative quantification of THP1 cell migration in response to CM from Mneo, Mxl90 and Mxl90 IKBSR cells. The quantification was performed by counting the number of migrated cells in at least 10 fields for each condition. **$p<0.01$.

Table 1. List of primers used for qRT-PCR.

GENE	FORWARD (5'-3')	REVERSE (5'-3')
CD163	CAGTCCCAAACACTGTCCTCGT	CAGGCGAAGTTGACCACTCTCTT
CD206	GCTGTTCTCCTACTGGACACCA'	AATCTGAGATTCGGACACCCA
CCL1	AAGAGCATGCAGGTACCCTTCT	CTCATTGGAGCAGATGGAGCT
CCL22	TGGCGTTCAAGCAACTGA	AAGRGTTTCACCACGCGCA
IL-10	GCGCTGTCATCGATTTCTTC	TGGCTTTGTAGATGCCTTTCTC
IL-12	GAGGCCTGTTTACCATTGGA	TCAAGGGAGGATTTTTGTGG
CD86	CACAGCAGAAGCAGCCAAAATG	TCTTCAGAGGAGCAGCACCAGA
HLA	GCCTCTTCTCAAGCACTGGGA	CCACCAGACCCACAGTCAGG
IL8	CACCGGAAGGAACCATCTCA	TGGCAAAACTGCACCTTCACA
IL1B	TGGCCCTAAACAGATGAAGTGC	CTGAAGCCCTTGCTGTAGTGGT
TNFA	GGAGGACGAACATCCAACCTT	CCCATTTCTCTTTTTGAGCCAG
MCSF	ATGCGCTTCAGAGATAACACCC	ATAGAAAGTTCGGACGCAGGC
CCL2	TCTCGCCTCCAGCATGAAAGT	GCATTGATTGCATCTGGCTGA
CCL5	TTTGTCACCCGAAAGAACCG	GAGGCAGAAACAGGCAAAT
ACTINA	ATTGCCGACAGGATGCAGAA	GCTGATCCACATCTGCTGGAA
IL1A	ATGAAGGTCGCATGGATCAATC	TCCCGTTGGCTACTACCAC
IL2	CAACTGGAGCATTTACTGCTGG	TCAGTTCTGTGGCCTTCTTGG
IL4	CCACGGACACAAGTGCGATAT	CTTGGAGGCAGCAAAGATGTCT
IL6	AAAGCAAAGAGGCACTGG	CCAGGCAAGTCTCCTCATTGAA
